# Understanding Adolescent Gynecological Issues: A Cross-Sectional Study at a Tertiary Care Center

**DOI:** 10.7759/cureus.57713

**Published:** 2024-04-06

**Authors:** Mukta Agarwal, Smita Singh, Chandra Jyoti, Shivangi Sinha, Simran Simran

**Affiliations:** 1 Obstetrics and Gynecology, All India Institute of Medical Sciences, Patna, Patna, IND; 2 Obstetrics and Gynecology, All India Institute of Medical Sciences, Bhubaneswar, Bhubaneswar, IND

**Keywords:** world health organization (who), pcod, teenager, gynaecological problems, menstrual problems, adolescents

## Abstract

Background and objectives: Adolescence is a crucial phase in a woman's life, as it signifies the beginning of her reproductive journey. During this time, there are notable variations in sexual development and a sense of caution that can present challenges for healthcare providers. The rationale for studying adolescent gynecological problems lies in the need to understand and address the unique reproductive health challenges faced by young girls. By investigating these issues, researchers and healthcare professionals can develop effective strategies for prevention, early detection, and treatment of gynecological conditions in adolescents. This knowledge is crucial for promoting the overall well-being and reproductive health of young girls, ensuring they receive appropriate care and support during this critical stage of development. This study focuses on identifying the most common gynecological issues in teenagers, exploring the root causes, examining the available treatment options, and understanding how they are managed at a tertiary care facility.

Methods: This cross-sectional observational study took place at a tertiary care center and focused on gynecological issues in adolescent females who sought care at the gynecology department from January 2016 to December 2022. The study participants were categorized into three groups: early adolescence (10-13 years), middle adolescence (14-16 years), and late adolescence (17-19 years) for analysis. Ethical approval was obtained for this hospital-based research, which involved examining, investigating, and treating the study participants.

Results: Out of the 49,700 new female patients, 2000 (4.02%) fell within the specified age range. The average age of the participants was 16.87±2.16, and the majority of them sought help for menstrual issues (63.45%), followed by abdominal discomfort (15.6%) and vaginal discharge (7.2%). Menstrual disorders were the most common concern, with dysmenorrhea and puberty menorrhagia being prevalent issues. Abdominal pain was caused by various factors, such as urinary tract infections, ovarian tumors, pelvic inflammatory disease, endometriosis, and endometrial tuberculosis. The majority of cases were treated conservatively, with only a small percentage requiring surgical intervention.

Conclusion: The significance of early detection and intervention in addressing gynecological issues among adolescents is highlighted in the findings of this research, underscoring the necessity for specialized healthcare services that cater to the specific needs of this demographic. Adolescent gynecology plays a crucial role in safeguarding the reproductive health and overall well-being of young women, emphasizing the importance of seeking assistance proactively.

## Introduction

The age range for adolescents, according to the World Health Organization (WHO), is between 10 and 19 years old [1]. India, with a teenage population of 253 million, has the highest number of teenagers in the world, with one in every five Indians falling into this age group [2]. Adolescence is a crucial stage of life that bridges the gap between childhood and adulthood, bringing about significant biological, physical, psychological, and social changes. Understanding and acknowledging these changes can help address the unique challenges faced by adolescents [3].

Among the various issues faced by this age group, menstrual irregularities are the most common complaint, often attributed to immaturity in the hypothalamic-pituitary-ovarian axis, leading to anovulation. Within 24 months after the onset of menstruation, 55% to 82% of teenagers establish a regular ovulatory cycle. However, even after this period, 22% of females continue to experience anovulation or irregular ovulation. In some cases, it may take up to five years after menarche for teenagers to achieve regular ovulatory cycles [4]. The primary cause of prolonged irregular menstruation is polycystic ovarian disorder (PCOD), which is the most prevalent endocrine disorder with long-term health implications [5]. Both PCOD and endometriosis can impact future fertility, emphasizing the need for prompt treatment [6].

Adolescents facing gynecological challenges often go unnoticed due to their reserved nature, resulting in a reluctance to share their concerns with their parents and seek medical help in a timely manner. Shockingly, even today, a staggering 42% of young girls in underdeveloped countries like India resort to using clothes instead of disposable sanitary products, putting them at a higher risk of developing pelvic inflammatory disease [2]. These young girls urgently need community-based education to promote safe menstrual practices and address the various health issues that may arise due to inadequate hygiene. As per the guidelines set by the American College of Obstetricians and Gynecologists (ACOG), adolescents must have their first visit to a gynecologist between the ages of 13 and 15, followed by annual check-ups to address their specific health concerns [7].

Adolescence is a critical phase in a young woman's life that necessitates a unique approach and careful attention. This transitional period exposes them to a wide range of challenges, including overall well-being, reproductive health, sexual topics, and psychological issues. It is during this time that young women prepare themselves for future motherhood, as they play a direct role in shaping the next generation. Excessive menstrual bleeding, also known as menorrhagia, significantly affects the overall quality of life at every stage. Effectively managing menorrhagia during puberty is crucial to preventing adolescents from developing anemia. Endocrine disorders such as polycystic ovary syndrome (PCOS) can have implications for both future reproductive and metabolic health. Therefore, it is essential to carefully assess and treat endocrine issues in adolescents for the sake of their reproductive well-being. 

The well-being of these adolescent girls not only impacts their health but also has a profound influence on the health of future generations. With this in mind, my research aims to delve into and address their various gynecological concerns and management strategies.

## Materials and methods

Study design: The study design for this research involved a single-center cross-sectional observational approach, which was carried out after obtaining approval from the Clinical Research and Ethics Committee at the All India Institute of Medical Sciences in Patna.

Settings: The study aimed to assess various gynecological disorders among teenage females (aged 10-19 years) at a tertiary care institution located in Bihar, a state in eastern India with a population of approximately 104.2 million people. The study spanned a duration of seven years, from January 2016 to December 2022, and was conducted after the ethics committee’s approval.

Participants: The participants included in the study were adolescent females between the ages of 10 and 19 who visited the outdoor clinic of the obstetrics and gynecology department. Informed written consent was obtained from each participant before their inclusion in the study. The participants were then categorized into three groups based on their age: early teenage (10-13 years), middle teenage (14-16 years), and late teenage (17-19 years). 

Study procedure: The study procedure involved gathering information from the participants regarding their specific concerns, such as pubertal changes, menstrual issues, vaginal discharge, pelvic discomfort, abdominal mass, obstetrical history (if married), and any other gynecological problems. Each participant underwent a clinical examination while ensuring privacy and confidentiality. Measurements such as height, weight, BMI, and secondary sexual traits were recorded. Additionally, blood tests and imaging studies were recommended as necessary. Once a proper diagnosis was made, a treatment plan was formulated. This study aimed to identify the most prevalent gynecological disorders in teenagers, investigate the underlying causes, examine current treatment choices, and comprehend how they are managed in a tertiary care setting.

Statistical methods: The statistical analysis of the data was performed using IBM SPSS Statistics for Windows, Version 25.0. The results for continuous measurements were presented as mean±SD, while categorical data was presented as frequency and percentage. The statistical software used for the analysis was developed by IBM Corp. and is located in Armonk, NY.

## Results

During the study period, a total of 38,000 new gynecological patients were enrolled, and a total of 11,700 pregnant females were enrolled (two days per week) in the gynecology department. Out of the 49,700 women involved in the study, 2,000 were between the ages of 10 and 19. Among these young women, 123 were pregnant, accounting for 6.15% of the group. Furthermore, 27 of them underwent abortions, making up 1.35% of the total. The remaining 1,850 experienced various gynecological issues, representing 92.5% of the group. 

The average age of the participants was 16.87 ± 2.16, with a range of 10 to 19 years. The majority of the participants were in their late teens, comprising 65.6% of the group. Mid-teens accounted for 24.9% of the participants, while early teens made up 9.4%. It is worth noting that the majority of the participants resided in urban areas, accounting for 56.9% of the group. Furthermore, 26.2% of the participants were married, indicating the presence of married adolescents seeking gynecological care. Additionally, 12.15% of the participants were illiterate, highlighting the need for education and awareness regarding reproductive health (Table 1).

**Table 1:** Socio-demographic details of study participants SD: Standard deviation, BMI: Body mass index

	Frequency (%)	Mean±SD
Age		
Early adolescence (10-13 years)	191 (9.6%)	12.03±1.12
Middle adolescence (14-16 years)	498 (24.9%)	15.25±0.77
Late adolescence (17-19 years)	1311 (65.6%)	18.18±0.75
Total	2000 (100%)	16.87±2.16
BMI		21.56±3.23
<18.5	405 (20.3%)	-
18.5-24.9	1275 (63.8%)	-
25-29.9	289 (14.5%)	-
>30	31 (1.6%)	-
Residence		
Rural	863 (43.2%)	-
Urban	1137 (56.9%)	-
Marital Status		
Married	523 (26.2%)	-
Unmarried	1477 (73.9%)	-
Education		
Illiterate	243 (12.15%)	-
Primary	580 (29%)	-
Secondary	687 (34.35%)	-
Higher Secondary	490 (24.5%)	-

The study conducted revealed a significant number of participants, totaling 1,269 individuals, who encountered various health issues, with menstrual problems being the most prevalent concern among them, making up 63.45% of the group. Additionally, abdominal discomfort was reported by 311 participants, representing 15.6% of the total, while 144 individuals dealt with vaginal discharge, accounting for 7.2% of the participants. Furthermore, less common health disorders such as breast lumps, infertility, lump abdomen, mastalgia, and backache were also identified, each affecting a smaller percentage of the group, as outlined in Table 2.

**Table 2:** Different presentations

	Early adolescence (10-13 years)	Middle adolescence (14-16 years)	Late adolescence (17-19 years)	Total
Menstrual problems	136	334	799	1269 (63.45%)
Pain abdomen	24	100	187	311(15.6%)
Vaginal discharge	14	33	97	144(7.2%)
Pregnant	0	7	116	123(6.15%)
Breast Lump	5	9	33	47 (2.35%)
Infertility	0	0	23	23 (1.15%)
Mastalgia	4	8	9	21 (1%)
Lump abdomen	6	5	14	25 (1.25%)
Missed abortion	0	0	16	16 (0.8%)
Incomplete abortion	0	0	11	11(0.5%)
Backache	2	2	6	10 (0.55%)
Total	191	498	1311	2000

Among the menstrual problems experienced by the participants, dysmenorrhea emerged as the most common issue, affecting 24.8% of women, followed by puberty menorrhagia at 23.95%, oligomenorrhea at 16.95%, and irregular menstruation at 14.5%. Notably, late adolescence was identified as the life stage with the highest prevalence of these menstrual problems, with 62.8% of participants affected, while middle adolescence accounted for 26.5% and early adolescence for 10.7%, as detailed in Table 3. These findings shed light on the distribution of health concerns across different age groups within the study population.

**Table 3:** Different menstrual problems in adolescents (N=1269)

Menstrual problems	Early adolescence (10-13 years)	Middle adolescence (14-16 years)	Late adolescence (17-19 years)	Total
Dysmenorrhea	40	91	184	315(24.8%)
Puberty menorrhagia	29	76	199	304(23.95%)
Oligomenorrhea	14	62	139	215(16.95%)
Irregular menstruation	22	38	124	184 (14.5%)
Primary amenorrhea	8	20	10	38 (3%)
Secondary Amenorrhea	14	18	83	115 (9%)
Hypomenorrhea	4	14	25	43 (3.4%)
Polymenorrhagia	2	10	16	28 (2.2%)
Premenstrual syndrome	3	5	19	27 (2.2%)
Total	136(10.7%)	334(26.5%)	799(62.8%)	1269

The data presented in the study underscores the importance of addressing menstrual health issues, as they appear to be a significant health concern among the participants. By identifying the specific problems faced by individuals, such as dysmenorrhea, menorrhagia, and irregular menstruation, healthcare providers can tailor interventions and support services to meet the unique needs of each group. Furthermore, the findings highlight the need for targeted health education and awareness programs aimed at promoting menstrual health and well-being, particularly among adolescents and young women who may be more vulnerable to these issues. 

Upon thorough evaluation of various menstrual problems, it was determined that dysmenorrhea was the most prevalent condition among the patients, affecting a significant number of individuals. The data revealed that out of 2000 patients, 315 were diagnosed with dysmenorrhea. Within this group, the majority, accounting for approximately 84.1%, experienced primary dysmenorrhea, while the remaining 15.88% were diagnosed with secondary dysmenorrhea. Notably, among those with secondary dysmenorrhea, 22 individuals were found to have endometriosis, shedding light on the diverse etiology of menstrual issues.

Following dysmenorrhea, puberty menorrhagia, and PCOD emerged as common concerns among the patients, impacting 304 and 290 individuals, respectively. Primary amenorrhea, characterized by the absence of menstrual periods, was linked to cervical or vaginal atresia in 26.3% of cases and constitutional delay in 2.6% of cases. On the other hand, secondary amenorrhea, where menstrual periods cease after being established, has a range of causes, including stress, PCOD, and premature ovarian failure, highlighting the complexity of menstrual health. 

Moreover, the analysis revealed that PCOD was a prevalent underlying cause of oligomenorrhea, a condition marked by irregular menstrual periods, affecting 64.2% of patients. Hypothyroidism was identified in 26.52% of cases, while hyperprolactinemia, low BMI, and endometrial tuberculosis were also found to contribute to oligomenorrhea in a smaller percentage of patients (Table 4). 

**Table 4:** Etiology of common menstrual problems 1: Dysfunctional menstrual bleeding, 2: Polycystic ovarian disorder, 3: Tuberculosis, 4: Idiopathic thrombocytopenic purpura, 5: Pelvic inflammatory disease, 6: Body mass index, 7: Mayer-Rokitansky-Küster-Hauser syndrome, 8: Obstructed hemivagina and ipsilateral renal anomaly

Puberty Menorrhagia (n=304)	
DUB^1^	226 (74.35%)
PCOD^2^	23 (7.5%)
Fibroid	21 (7%)
Hypothyroidism (TSH>4.0 mU/L)	11 (3.6%)
Endometrial TB^3^	10 (3.3%)
ITP^4^	8 (2.64%)
Hyperprolactinemia (Serum Prolactin>25 ng/ml)	5 (1.7%)
Dysmenorrhea (N=315)	
Primary Dysmenorrhea	265 (84.1%)
Secondary Dysmenorrhea	50 (15.88%)
PID^5^	13 (4.1%)
Endometriosis	22 (7%)
Ovarian cyst	8 (2.5%
Uterine anomaly	7 (2.3%)
Oligomenorrhea (N=215)	
PCOD	138 (64.2%)
Hypothyroidism (TSH<4)	57 (26.52%)
Hyperprolactinemia (Serum Prolactin>25ng/ml)	11 (5.12%)
Low BMI^6 (<18.5)^	7 (3.26%)
Endometrial TB	2 (1%)
Primary amenorrhea (N=38)	
Cervical/vaginal atresia	10(26.3%)
MRKH^7^	9 (23.7%)
Transverse Vaginal septum	6 (15.8%)
OVHIRA^8^	6 (15.8%)
Gonadal dysgenesis	2 (5.2%)
Imperforate hymen	2 (5.3%)
Testicular feminising syndrome	2 (5.3%)
Constitutional delay	1 (2.6%)
Secondary Amenorrhea (N=115)	
PCOD	44 (38.2%)
Stress	25 (21.8%)
LOW BMI (<18.5)	20 (17.4%)
Hypothyroidism (TSH<4)	13 (11.3%)
Endometrial TB	10 (8.7%)
Premature ovarian failure	3 (2.6%)

This comprehensive examination of menstrual problems underscores the importance of understanding the diverse etiology behind these conditions and the need for tailored treatment approaches to address the specific underlying causes. By identifying and addressing the root causes of menstrual issues, healthcare providers can offer more effective management strategies and improve the overall well-being of individuals experiencing these concerns.

**Figure 1:**
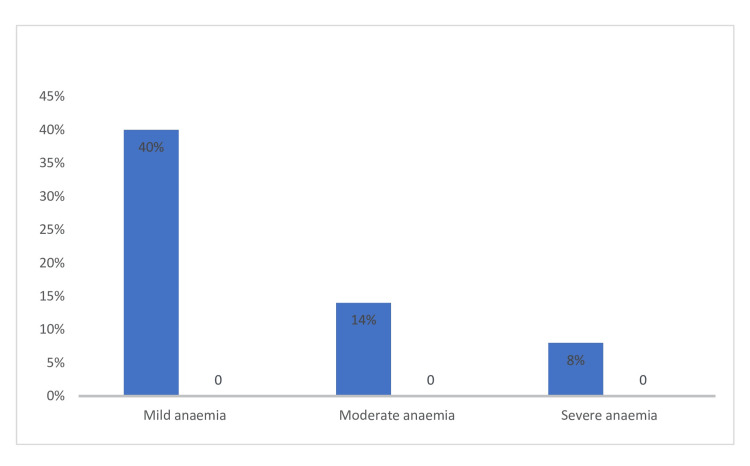
Prevalence of anemia in adolescent females Mild anemia-hemoglobin (Hb): 10-10.9 gm/dl, Moderate anemia: Hb 7-9.9 gm/dl, Severe anemia: Hb < 7 gm/dl

Discover the alarming statistics: Anemia was detected in 1243 individuals, making up 62.15% of the cases. The breakdown revealed that 40% had mild anemia, 14% had moderate anemia, and 8% had severe anemia. Hemoglobin levels of 10-10.9 gm/dl were classified as mild, 7-9.9 gm/dl as moderate, and less than 7 gm/dl as severe (Figure 1).﻿

Discover the truth behind teenagers with PCOD: An astonishing 67.25% of cases were detected during the later stage of adolescence, shedding light on the prevalence of this condition among young individuals. Moreover, it was found that 29.7% of these teenagers were either overweight or obese, while 11% were underweight. Interestingly, the majority, accounting for 59.3%, had a normal BMI falling within the range of 18.5 to 25. Various symptoms were reported, including menorrhagia (excessive menstrual bleeding) affecting 8% of cases, hypomenorrhea (scanty menstrual flow) affecting 4.8%, oligomenorrhea (infrequent menstrual periods) affecting 47.6%, irregular menses affecting 16.9%, secondary amenorrhea (absence of menstruation) affecting 15.2%, and infertility affecting 7.5%. Additionally, hirsutism (excessive hair growth) was observed in 21.38% of individuals, while acne affected 29.7% of the cases. The majority of cases, approximately 72.4%, exhibited polycystic ovaries, a characteristic feature of PCOD. Furthermore, 23.5% of the teenagers had impaired glucose tolerance, 11% had elevated serum testosterone levels, and 8.6% had increased fasting insulin levels. In terms of treatment, lifestyle changes were recommended for 60.35% of cases, emphasizing the importance of adopting healthy habits. On the other hand, hormonal therapy was prescribed for 39.65% of cases, highlighting the need for medical intervention (Table 5).

**Table 5:** Adolescents with PCOD (N=290) USG: Ultrasonography

Weight	
Obese (BMI>25)	86 (29.7%)
Underweight (BMI<18.5)	32 (11%)
Normal BMI (18.5-25)	172 (59.3%)
Age	
Early (10-13 years)	21 (7.25%)
Middle (14-16 years)	74 (25.5%)
Late (17-19 years)	195 (67.25%)
Presentation	
Oligomenorrhea	138 (47.6%)
Irregular menses	49 (16.9%)
Secondary Amenorrhea	44 (15.2%)
Hypomenorrhea	14 (4.8%)
Puberty Menorrhagia	23 (8%)
Infertility	22 (7.5%)
Polycystic ovaries on USG	210 (72.4%)
Clinical and biochemical changes	
Acne	86 (29.7%)
Hirsutism	62 (21.38%)
Raised fasting insulin (>20 μU/mL)	25 (8.6%)
Raised serum testosterone (> 20 ng per dL)	32 (11%)
Impaired glucose tolerance (glucose level between 140-199 gm/dl after 2-hour 75 gm glucose challenge)	68 (23.5%)
Treatment	
Lifestyle modification	175 (60.35%)
Hormonal treatment	115 (39.65%)

**Figure 2:**
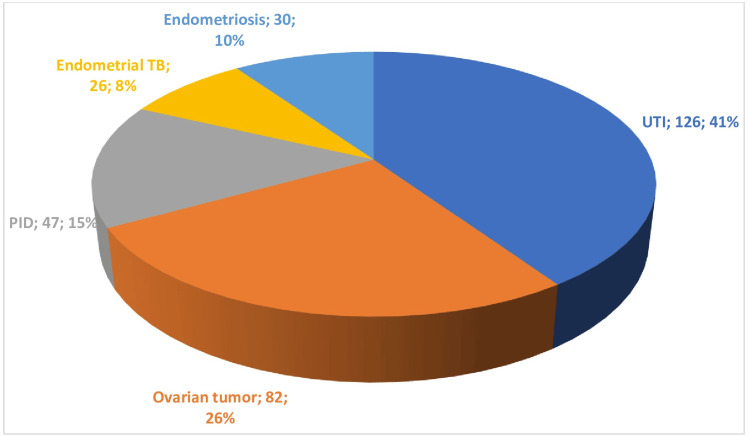
Causes of pain abdomen (n=311/2000)

Out of the 2000 participants in the study, 311 individuals, representing 15.6% of the sample, reported experiencing abdominal pain. The causes of this pain varied among the participants, with urinary tract infections (UTIs) being the most common at 41%, followed by ovarian tumors at 26%, pelvic inflammatory disease (PID) at 15%, and endometriosis and endometrial tuberculosis at 10% and 8%, respectively. These findings demonstrate the diverse range of conditions that can lead to abdominal pain (Figure 2).

**Figure 3:**
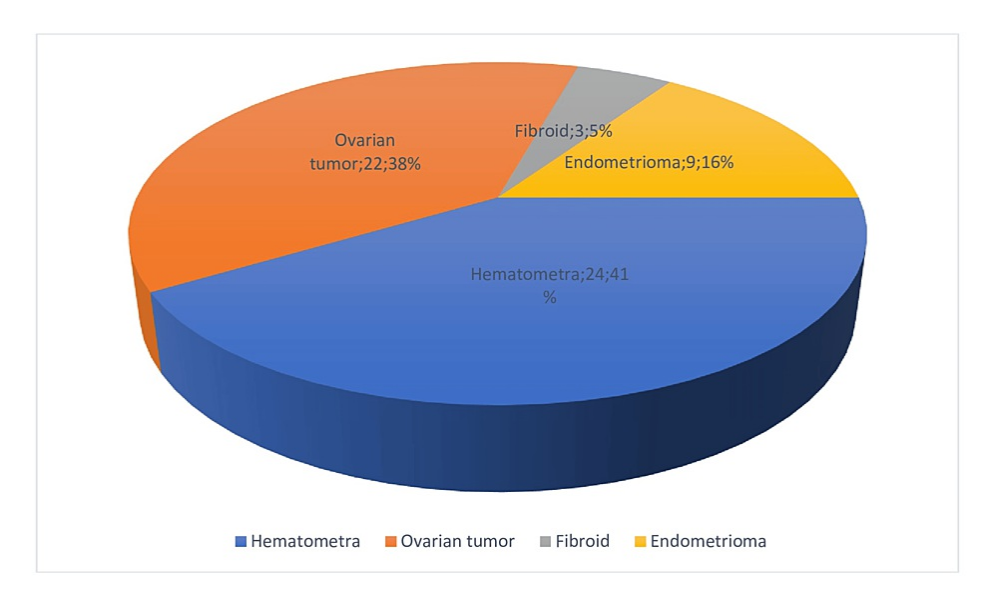
Different etiology of lump abdomen (n=58/2000)

Among the teenagers in the study, 58 individuals, or 2.9% of the participants, were found to have abdominal lumps. Further investigation revealed that these lumps were due to hematometra, ovarian tumors, endometrioma, and fibroids. Early detection and diagnosis are crucial in teenagers, as abdominal lumps can indicate underlying medical conditions that require attention (Figure 3).

**Figure 4:**
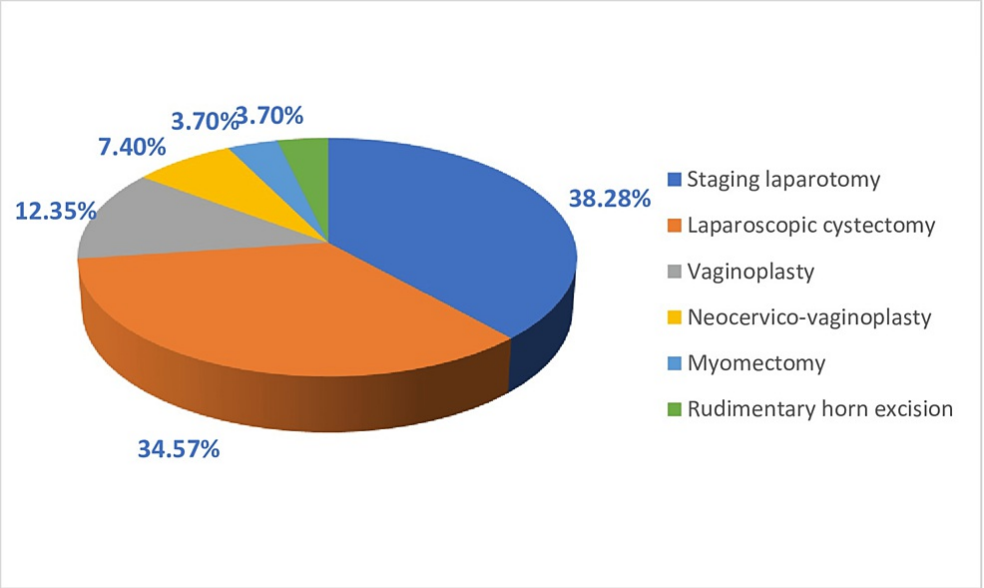
Different surgical management was offered (n=81/2000 [4%])

Except for 81 patients, nearly all participants received medical or conservative care for their conditions. Various surgical procedures were performed to address abdominal issues, including staging laparotomy, laparoscopic cystectomy, vaginoplasty, neocervico-vaginoplasty, myomectomy, and rudimentary horn excision, with percentages of 38.28%, 34.57%, 12.35%, 7.4%, and 3.7%, respectively (Figure 4).

These interventions underscore the importance of personalized treatment plans tailored to each patient's specific needs. The study results offer valuable insights into the prevalence, causes, and treatment strategies for abdominal pain, particularly in teenagers, and can help enhance healthcare practices in this area.

## Discussion

In 2010, the sample registration system statistical report revealed that 10% of the Indian population consists of adolescent females [8]. To cater to this demographic, the Ministry of Health and Family Welfare launched Rashtriya Kishor Swasthya Karyakram (RKSK) on January 7, 2014, to reach 253 million teenagers. The program highlights the importance of establishing Adolescent Friendly Health Clinics (AFHC) to offer information and counseling on various adolescent health issues [9].

In the present investigation, 4.02% of female patients visiting the gynecology department were in their adolescent years, while Lalitha et al. and Pegu et al. reported percentages of 3.41% and 8.33%, respectively. The most commonly reported gynecological problems among these patients were menstrual issues (63.45%), abdominal pain (15.6%), vaginal discharge (7.2%), adolescent pregnancy (6.15%), and infertility (1.15%). These findings align with Lalitha et al.'s study, which identified menstrual disorders (60%), vaginal discharge (10.66%), teenage pregnancy (10.66%), and infertility (2.66%) as the most prevalent issues among adolescent girls [10]. Pegu et al. also found menstrual disorders (67.11%) to be the primary gynecological condition in adolescent females, followed by vaginal discharge (18.68%), urinary tract infection (9.8%), and teenage pregnancy (0.84%) [11]. In a cross-sectional study conducted by Geeta et al. in the Lucknow district of India, 600 adolescents were interviewed, revealing a mean age of menarche at 13.1 years, with 35.3% experiencing menstrual problems, 86.5% reporting dysmenorrhea, and 31% having a vaginal discharge [12]. Similarly, a study in Nigeria involving 482 adolescents found a mean age of menarche at 13.13±1.37 years, with 6.0% experiencing irregular cycles. Premenstrual syndrome and dysmenorrhea were the major menstrual issues, affecting 14.3% and 12.2% of the participants, respectively [13]. Another study reported a prevalence of 33.4% for menstrual irregularity [14].

Our findings indicate that the majority of teenagers, accounting for 65.6%, are in the late adolescence stage, specifically between the ages of 16 and 19. These teenagers primarily reside in urban areas, making up 56.9% of the population. It is worth noting that 26.2% of them are married, while 12.15% are illiterate. According to the National Family Health Survey (NFHS-4), more than three million females are married before the legal age of 18, and 370,000 girls experience teenage pregnancy [15]. Our research also reveals that the average age is 16.87±2.16, the average age of menarche is 12.2, and the mean BMI is 21.56±3. These findings align with the De Sanctis et al. study, which reported an average age of 17.1±1.4, an average age of menarche of 12.4±1.3, and a mean BMI of 20.32±2 [16]. In comparison to the research conducted by Kalyankar et al., who found that 14.6% of individuals were overweight, 3.4% were obese, and 26% were underweight, our study reveals that 14.5% and 1.6% of individuals are overweight and obese, respectively, while 20.3% are underweight [17].

In late adolescence, menstrual disorders were found to be most common, affecting 66.2% of individuals. The leading issues were dysmenorrhea and puberty menorrhagia, with a prevalence of 24.8% and 23.95%, respectively. Anaemia was identified in 62.15% of patients, with 8% requiring a blood transfusion due to severe anemia. A study by Cassava et al. revealed that 63.8% of individuals with menstrual problems were in their late adolescence years, and anemia was prevalent in 64.5%, with 7.7% having severe anemia [18]. Other common issues in this age group include dysmenorrhea and persistent pelvic discomfort. The prevalence of dysmenorrhea ranges from 30% to 89%, with severe cases observed in 14% to 23% of cases [19,16,20].

Recent research has also shown that painful cycles can occur in non-ovulatory cycles as well [21]. Persistent pelvic discomfort can be associated with gynecological issues such as endometriosis, outflow tract blockage, ovarian cyst or mass, and pelvic inflammatory disease. In our study, 84.1% of patients had no definitive cause for dysmenorrhea, while secondary dysmenorrhea was primarily caused by endometriosis (7%), which aligns with previous studies [22,23].

In our research, we found that primary amenorrhea, which is the absence of menstruation in girls who have reached the age of 16, had various causes. In 26.3% of cases, it was attributed to cervical or vaginal atresia, which is the abnormal closure or absence of the cervix or vagina. Another 2.6% of cases were due to constitutional delay, which refers to a normal variation in the timing of puberty. When it came to secondary amenorrhea, which is the absence of menstruation in women who have previously had regular periods, we discovered different causes. The most common cause, accounting for 38.2% of cases, was stress. Polycystic ovary syndrome (PCOS) was responsible for 21.8% of cases, while premature ovarian failure accounted for 2.6% of cases. Interestingly, another study presented different findings regarding the etiology of primary amenorrhea. They reported that cervicovaginal atresia was the leading cause, accounting for 26.3% of cases. Mayer-Rokitansky-Küster-Hauser (MRKH) syndrome, a condition characterized by the underdevelopment or absence of the uterus and vagina, was responsible for 23.7% of cases. Transverse vaginal septum, a condition where a wall divides the vagina, accounted for 15.8% of cases. Obstructed Hemi-vagina and Ipsilateral Renal Anomaly (OVHIRA), a rare condition where one side of the vagina is blocked and is associated with a kidney abnormality also accounted for 15.8% of cases. Constitutional delay was the least common cause, seen in only 2.6% of cases [24,25]. In a separate study conducted by Chandrakala et al., the most common cause of primary amenorrhea was found to be gonadal dysgenesis, which refers to the abnormal development of the ovaries, and it accounted for 50% of the participants. Imperforate hymen, a condition where the hymen completely blocks the vaginal opening, was the cause in 33% of cases, while vaginal atresia accounted for 17% of cases [26]. 

These findings highlight the diverse range of causes for both primary and secondary amenorrhea, emphasizing the importance of thorough evaluation and individualized treatment approaches for each patient. In our research, we found that the most common cause of oligomenorrhea among the participants was polycystic ovary syndrome (PCOS), accounting for 138 out of 215 cases. Following PCOS, the next, leading causes were hypothyroidism, with 57 cases, and hyperprolactinemia, with 11 cases. Interestingly, we observed that a low body mass index (BMI) and endometrial tuberculosis were rarely associated with oligomenorrhea, accounting for only 7 and 2 cases out of 215, respectively. 

When we examined a larger sample size of 2000 adolescents, we discovered that PCOS was prevalent in 15% of them, which is consistent with previous studies. Among those with PCOS, 29.7% were overweight or obese, indicating a potential link between PCOS and weight-related issues. On the other hand, 11% of the adolescents with PCOS were underweight, suggesting that weight can vary among individuals with this condition. Furthermore, the majority of adolescents with PCOS, approximately 67.25%, were in their late teens. The symptoms reported by the participants with PCOS were diverse. Oligomenorrhea, characterized by infrequent menstrual periods, was the most common symptom, affecting 47.6% of the individuals. Other symptoms included irregular menstruation (16.9%), secondary amenorrhea (15.2%), puberty menorrhagia (excessive menstrual bleeding during puberty) (8%), infertility (7.5%), and hypomenorrhea (abnormally light menstrual flow) (4.8%). Additionally, we observed that 72.4% of the patients with PCOS had polycystic ovaries, a characteristic feature of the syndrome. Alongside this, acne was present in 29.7% of the patients, hirsutism (excessive hair growth) in 21.38%, and poor glucose tolerance in 23.5%. Our findings align with a study conducted by Hickey et al., which reported a prevalence of PCOS in 6 to 13% of teenage girls. The study also revealed that 21% of the girls with PCOS were overweight, while 8% were obese. Furthermore, 35.4% of the participants had polycystic ovaries detected through ultrasound, 51.7% experienced menstrual irregularities, and 48% had minor acne. These similarities between our research and Hickey et al.'s study provide further support for the prevalence [27]. 

The majority of issues faced by adolescents were addressed through conservative management, with only a small percentage (4%) requiring surgical intervention for conditions such as ovarian tumors, outflow tract obstruction, and myoma. The surgical procedures most frequently performed were staging laparotomy followed by laparoscopic cystectomy. In cases of outflow tract obstruction, vaginoplasty was the chosen surgical approach [28-30].

The data presented in the study highlights the importance of individualized treatment plans for adolescents facing various health issues. By opting for conservative management in the majority of cases, healthcare providers can prioritize the well-being and long-term health outcomes of young patients. This approach not only minimizes the need for invasive procedures but also underscores the importance of careful consideration and evaluation before resorting to surgical intervention. In instances where surgery was deemed necessary, the specific procedures mentioned in the study, such as staging laparotomy and vaginoplasty, were chosen based on the unique needs of each patient and the nature of their condition. This tailored approach ensures that adolescents receive the most appropriate care and treatment for their specific health concerns.

Implications and contribution: Throughout seven years, an in-depth examination was undertaken to explore a myriad of difficulties faced by teenagers, with a special emphasis on menstrual issues, which emerged as the most prevalent concern. With only a few isolated cases, the vast majority of illnesses were effectively managed through conservative methods. By acquiring a comprehensive understanding of the common gynecological challenges encountered by adolescents, this study undoubtedly serves as a guiding light for future healthcare professionals, enabling them to provide optimal treatment and effectively address the hurdles in adolescent healthcare.

Strengths and limitations: However, the study's limitation lies in its single-center design, which may raise questions about the generalizability of the results to other settings or populations. While the findings may be robust within the specific context of the tertiary care center where the study was conducted, they may not necessarily apply to different healthcare facilities or patient populations. Future research could benefit from including multiple centers to ensure that the results are more widely applicable and can be generalized to a broader population.

## Conclusions

Adolescent females often face a range of gynecological issues, with menstrual problems being particularly common. These issues require special attention and care from healthcare professionals. By implementing a thorough evaluation process, medical experts can accurately diagnose any underlying problems and provide the necessary treatment. This approach not only addresses the immediate concerns but also takes into consideration the long-term well-being and fertility preservation of these young individuals. It is essential to adopt a holistic approach to managing gynecological problems in adolescents, combining conservative measures and surgical interventions when needed to achieve optimal outcomes and ensure the overall health and happiness of these young women.

## Figures and Tables

**Figure 5:**
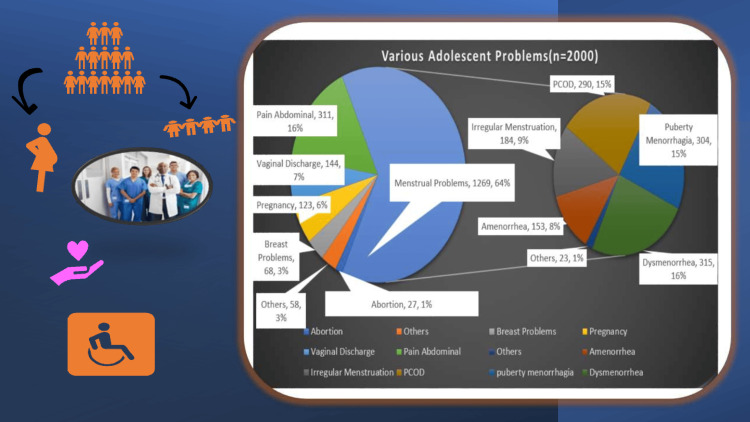
Graphical representation of study
